# Napoleon Cybulski (1854–1919)

**DOI:** 10.1007/s00415-013-6863-9

**Published:** 2013-02-13

**Authors:** Andrzej Grzybowski, Krzysztof Pietrzak

**Affiliations:** 1 Department of Ophthalmology, Poznan City Hospital, Ul. Szwajcarska 3, 61-285 Poznań, Poland; 2 Department of Ophthalmology, University of Warmia and Mazury, Olsztyn, Poland; 3 Department of Orthopaedics and Traumatology, University of Medical Sciences, Poznań, Poland


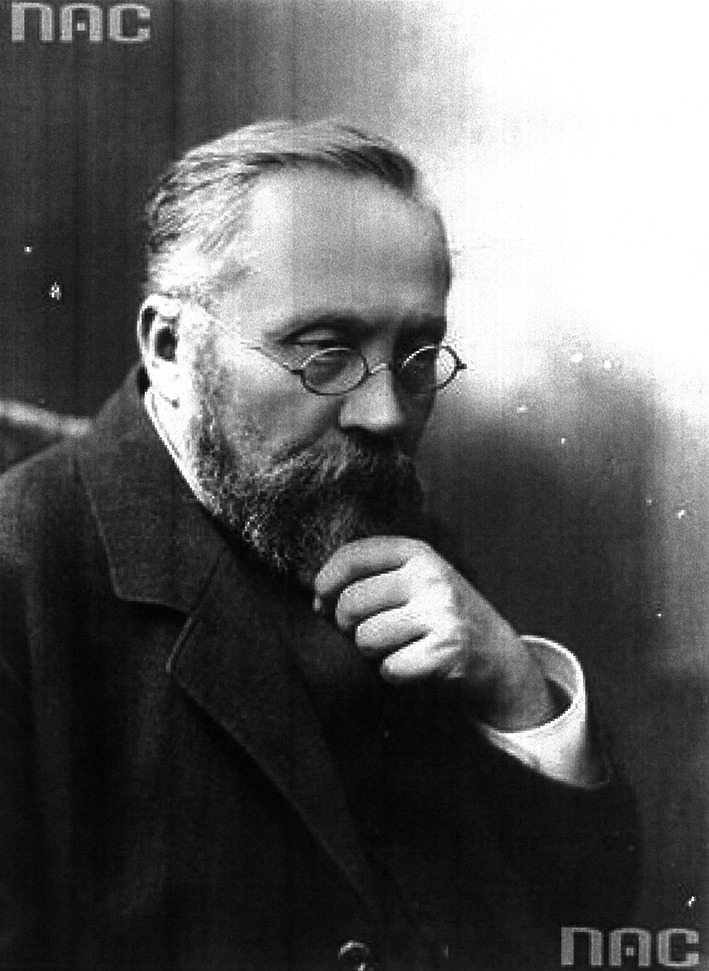
 Napoleon Cybulski was born on September 14, 1854, in Krzywonose, which was then in Polish territory under Russian tsaristic rule. He came from a noble family. After grammar school in Minsk, he started medical studies in Petersburg at the Military Medical Academy. In 1880 he received a diploma in medicine *cum exima laude* (with the highest distinction). From 1877 to 1885 he was an assistant at the Institute of Physiology. He obtained a doctorate in 1885 [2], with a thesis on the velocity of blood flow as detected by an apparatus called *photohematochrometer*, of his own construction. He also conducted research on the influence of the phrenic nerve on the respiration rate, and on the larynx and vagus nerves.

In 1885 he was offered the position of chairman at the Institute of Physiology at the Jagiellonian University, Kraków (now Poland, then part of the Austro-Hungarian Empire). There he was dean of the Medical Faculty, and subsequently rector of the University. In Kraków his scientific career blossomed. In 1895 he isolated the active factor from suprarenal tissue: *nadnerczyna*, later called adrenalin [1]. He measured and described the velocity of the blood flow in femoral and cervical arteries. He also found that an increase in intracranial pressure causes disturbances in blood flow to the brain [8].

Remaining in the same position, he continued his neurological research. Under Cybulski’s supervision, Adolf Beck (1863–1942) started pioneer studies on the activity of the cerebral cortex in response to peripheral nerve stimulation in dogs and monkeys. Electrodes were placed on the skull to record the changes in the electric potential [5]. In this way they invalidated William Horsley’s notion that these changes reflected activity of muscles of the skull. By further analyses of potential changes, they mapped out sensory regions of cerebral cortex. They also showed that the amplitude of the signal depended on the strength and kind of sensory stimulus and on the depth of anesthesia. They suspected that brain function was mediated by bioelectrical activity of neurons. Their studies on brain mapping and nerve stimulation were absolutely innovative, since they were not familiar with earlier research by Richard Caton on changes in bioelectrical activity of the dog brain during to sleep, activity and changes in behaviour. Again in cooperation with Beck, Cybulski showed that every taste sensation in the tongue was caused by a separate kind of receptor. He described the difference between afferent and efferent impulses entering and leaving the spinal cord on the basis of recordings from dorsal and ventral roots [10].

Cybulski also studied the bioelectrical activity of muscles. He used electrical stimulation to study the pathway of an electrical current through muscles, conducting his research using a capacitor of his own construction. Its construction allowed him to make the process of electric irritation very consistently and accurately [3]. Cybulski described the ‘resting current’ as a difference in potential between the interior and exterior of muscle cells caused by two different groups of ions [6]. Today, Cybulski’s *resting current* is called the resting potential. By explaining that this resting potential difference was the normal state of all muscle cells, he refuted Hermann’s idea that the difference resulted from cell damage. Moreover, he was the first to explain that the division into two groups of ions is caused by different permeability of membranes for various positive and negative ions. That was the new idea which completed and expanded the experiments of Du Bois Reymond and others. He used theoretical and experimental methods on an artificial model of muscle. He suspected that the ion movement caused the ‘active current’—in other words, that the electrical signals are caused by the chemical ones. To prove it, he constructed a model of muscle from a frog’s bowel membrane. By using solutions with different ion concentrations on either side of the membrane, he obtained different resting currents. Cybulski concluded that the resting potential was caused by negative ions inside and positive ions outside the muscle membrane, and that the active current was the effect of positive ion movement from the outside to the inside [6, 7]. He also indicated that temperature had a marked influence on the process. Cybulski also studied the active current in muscle in relation to the force and character of stimulation by means of two electrodes attached to muscle tissue, as well as the influence of the distance between the two stimulating electrodes on the active current. His observations later evolved into electromyography and the study of nerve conduction.

Some ideas from Cybulski’s book about hypnosis [4] were very brave and preceded Freud’s notion of the unconscious.

Between 1913 and 1914, Cybulski again studied the bioelectrical activity of the brain and found changes in the amplitude and rate of cortical electrical activity during an induced seizure [9]. It was 15 years before Berger would discover the EEG and the alpha rhythm.

He was a very loving and humble person, infecting others with his scientific enthusiasm. He authored more than 100 scientific papers and was the spiritual father of several prominent physiologists who started their careers in other Polish academic centres. Cybulski was a declared protagonist of medical education for women, and in 1891 he established the first gymnasium for girls in Kraków. He was also a member of the Kraków city council. He ran a dental surgery office to meet the financial needs of his large family and his wife Julia Rogozinska. He died of a stroke on April 26, 1919, in his university office in Kraków.
